# Impact of Chronic HIV Infection on Acute Immune Responses to SARS-CoV-2

**DOI:** 10.1097/QAI.0000000000003399

**Published:** 2024-02-26

**Authors:** Skye Opsteen, Tim Fram, Jacob K. Files, Emily B. Levitan, Paul Goepfert, Nathaniel Erdmann

**Affiliations:** a Division of Infectious Diseases, Department of Medicine, University of Alabama at Birmingham, Birmingham, AL; and; b Department of Epidemiology, University of Alabama at Birmingham, Birmingham, AL.

**Keywords:** SARS-CoV-2, acute COVID-19, people living with HIV, immune response, inflammation

## Abstract

There is mounting evidence that HIV infection is a risk factor for severe presentations of COVID-19. We hypothesized that the persistent immune activation associated with chronic HIV infection contributes to worsened outcomes during acute COVID-19. The goals of this study were to provide an in-depth analysis of immune response to acute COVID-19 and investigate relationships between immune responses and clinical outcomes in an unvaccinated, sex- and race-matched cohort of people with HIV (PWH, n = 20) and people without HIV (PWOH, n = 41). We performed flow cytometric analyses on peripheral blood mononuclear cells from PWH and PWOH experiencing acute COVID-19 (≤21-day postsymptom onset). PWH were younger (median 52 vs 65 years) and had milder COVID-19 (40% vs 88% hospitalized) compared with PWOH. Flow cytometry panels included surface markers for immune cell populations, activation and exhaustion surface markers (with and without SARS-CoV-2–specific antigen stimulation), and intracellular cytokine staining. We observed that PWH had increased expression of activation (eg, CD137 and OX40) and exhaustion (eg, PD1 and TIGIT) markers as compared to PWOH during acute COVID-19. When analyzing the impact of COVID-19 severity, we found that hospitalized PWH had lower nonclassical (CD16^+^) monocyte frequencies, decreased expression of TIM3 on CD4^+^ T cells, and increased expression of PDL1 and CD69 on CD8^+^ T cells. Our findings demonstrate that PWH have increased immune activation and exhaustion as compared to a cohort of predominately older, hospitalized PWOH and raises questions on how chronic immune activation affects acute disease and the development of postacute sequelae.

## Abstract 2

Supplemental Digital Content is Available in the Text.

## INTRODUCTION

Most individuals infected with severe acute respiratory syndrome coronavirus 2 (SARS-CoV-2) experience self-limited disease and recover within 1–2 weeks. However, a portion of individuals experience more severe illness, associated with hospitalization, increased oxygenation requirements, and mortality.^1–7^ Although the frequency of severe SARS-CoV-2 presentations has decreased from its peak because of increased population immunity, high rates of transmission continue to trigger severe illness in patients with impaired immunity.^8–13^ Although intensive efforts have characterized the immune responses and associated clinical outcomes during acute COVID-19, the majority of this work has focused on immunocompetent individuals.

Early in the COVID-19 pandemic, there was concern that HIV infection may predispose individuals to more severe presentations in acute COVID-19 because of impaired immune function and the persistent inflammation driving premature aging in people with HIV (PWH).^14–19^ Recent studies have shown PWH have more severe SARS-CoV-2 clinical courses and higher mortality rates, although this may be attributable to lack of viral suppression, the higher prevalence of comorbidities seen in PWH, or the chronic inflammatory state in HIV.^20–24^ To date, limited studies have evaluated the immune response to acute COVID-19 in PWH. Increased expression of activation and exhaustion markers as compared to people without HIV (PWOH) have been reported,^23,25^ but these studies were limited to few markers (human leukocyte antigen [HLA]-DR, PD1, and TIM3) as compared to PWOH.^6,26–34^ We sought to address this knowledge gap by performing an in-depth analysis of immune responses during acute COVID-19 in a cohort of PWH with varying levels of COVID-19 disease severity. We hypothesized the chronic inflammatory changes associated with HIV would amplify the immunologic sequelae of acute SARS-CoV-2 infection and increase the likelihood of developing severe disease. We provide a comprehensive look at immune responses during acute COVID-19 in PWH, including characterization of the activation and exhaustion phenotype of CD4^+^ and CD8^+^ T cells and early SARS-CoV-2–specific T-cell responses, as well as demonstrate how these findings were affected by COVID-19 severity.

## METHODS

### Sample Collection

Participants with or without diagnostic codes indicating HIV infection were identified and matched based on sex and race. HIV status was confirmed based on review of medical records. Peripheral blood was collected from individuals with confirmed acute SARS-CoV-2 infection with (HIV + COVID, n = 20) or without (COVID, n = 41) confirmed chronic HIV infection at the University of Alabama at Birmingham. All participants in the HIV + COVID cohort were virally suppressed on antiretroviral therapy (ART). Specimens were collected during acute SARS-CoV-2 infection (defined as ≤21-day postsymptom onset and confirmatory SARS-CoV-2 testing) when the Alpha variant was the dominant circulating variant (defined as before July 1, 2021). No participants were vaccinated before SARS-CoV-2 infection. Peripheral blood mononuclear cells (PBMCs) were isolated as previously described.^34^ This study was approved by the Institutional Review Board at the University of Alabama at Birmingham, and appropriate consent was received for all participants included in the study.

Clinical and demographic information were collected for all participants (Table 1). Clinical metrics were obtained retroactively to avoid sampling bias. Medical records were used to collect SARS-CoV-2–specific metrics including symptom onset, hospitalization status, illness severity, and medications prescribed for SARS-CoV-2 symptoms. Illness severity definition was based on the World Health Organization's Clinical Progression Scale^35^: (1) nonhospitalized (categories 1–2), (2) mild-moderate illness (categories 3–4), (3) severe illness (categories 5–7), and (4) mortality (category 8). HIV-related characteristics were also obtained from the medical record (see Table, Supplemental Digital Content 1, http://links.lww.com/QAI/C235, HIV-related characteristics).

**TABLE 1.** Cohort Demographics

	HIV + COVID	COVID	*P*
Sample size	20	41	
Age^*^	52 (30–76)	65 (25–93)	0.001§
Sex^†^			0.319
Female	9 (45%)	24 (59%)	
Male	11 (55%)	17 (42%)	
Race^†^			0.796
AA	12 (60%)	26 (63%)	
W	8 (40%)	15 (37%)	
Days postsymptom onset^*^	7 (1–20)	4 (0–16)	0.052
Disease severity^†,‡^			<0.001§
Nonhospitalized	12 (60%)	5 (12%)	
Mild-moderate illness	1 (5%)	23 (56%)	
Severe illness	6 (30%)	7 (17%)	
Deceased	1 (5%)	6 (15%)	
Comorbidities^†^			
Hypertension	16 (80%)	34 (83%)	>0.999
Obesity	9 (45%)	21 (51%)	0.786
Diabetes	7 (35%)	22 (54%)	0.187
COPD	6 (30%)	8 (20%)	0.518
Cancer	4 (20%)	8 (20%)	>0.999
ART regimen			
NRTI	19 (95%)		
NNRTI	0 (0%)		
PI	4 (20%)		
INSTI	18 (90%)		
CD4			
Percent	32% (13%–45%)		
Count	729 (181–1619)		

Statistical analysis was performed for each category.

* Mann–Whitney *U* test.

† Fisher exact test.

‡ Analysis compared frequency of nonhospitalized with hospitalized individuals.

Percentages were rounded to the nearest whole number.

AA, African American; COPD, chronic obstructive pulmonary disease; INSTI, integrase strand transfer inhibitor; NNRTI, non-nucleoside reverse transcriptase inhibitor; NRTI, nucleoside reverse transcriptase inhibitor; PI, protease inhibitor; W, White.

§ *p* ≤ 0.05.

### Flow Cytometric Phenotypic Analyses

Isolated PBMCs were thawed in R-10 media, RPMI (Gibco) with 10% human serum AB (GeminiBio), and then stained using 2 panels. Immune cell subsets were identified using the following panel: CD3-APC-eFluor 780, CD4-BV711, CD8-V500, CD14-Alexa Fluor 700, CD16-FITC, CD19-BUV563, CD19-BUV563, CD27-PE/Dazzle 594, CD28-APC, CD38-BUV737, CD45-PE-Cy7, CD56-BV421, CD57-PerCP-Cy5.5, and HLA-DR-PE. T-cell surface markers for activation/exhaustion were further characterized using the following panel: CD3-APC-eFluor 780, CD4-PE-eFluor 610, CD8-FITC, CD14-BUV563, CD19-BUV563, OX40-Pe-Cy7, CD69-BUV737, CD137-BV650, CD154-APC, PD1-BV785, TIGIT-Alexa Fluor 700, PDL1-PE, and TIM-3-BV421 (see Table, Supplemental Digital Content 2, http://links.lww.com/QAI/C236, Flow cytometry panels). All panels used Live/Dead-UV blue (Invitrogen) to exclude dead cells from analysis. Samples were fixed with a 1% formalin solution and stored at 4°C for no more than 72 hours until analysis on a Symphony A3 (BD Biosciences) flow cytometer machine. Gates were set by conducting an unstimulated negative control test with equimolar concentration of dimethyl sulfoxide and a positive control test with Staphylococcal enterotoxin B (SEB, 1 µg/mL) (Toxin Technology) in parallel with all samples (see Figure, Supplemental Digital Content 3, http://links.lww.com/QAI/C237, Figure, Supplemental Digital Content 4, http://links.lww.com/QAI/C238, Gating strategy). Results were analyzed using FlowJo v10 (BD Biosciences) software.

### Activation-Induced Markers and Intracellular Cytokine Staining Assays

PBMCs were thawed and rested for 6–7 hours before stimulation with 1 of 2 peptide pools (JPT Peptide Technologies) at 1 µL/mL per peptide: (1) SARS-CoV-2 Spike glycoprotein peptide pool (S pool) containing 315 (15-mers with 11-amino acid overlap) peptides (PM-WCPV-S-1); (2) SARS-CoV-2 mega pool (M pool) containing 16 peptides (15-mers with 11-amino acid overlap) from Envelope protein (PM-WCPV-VEMP-1), 102 peptides (15-mers with 11-amino acid overlap) from Nucleoprotein (PM-WCPV-NCAP-1), and 53 peptides (15-mers with 11-amino acid overlap) from Membrane protein (PM-WCPV-VME-1).

For Activation-Induced Markers (AIM), cells were cocultured with CD154-APC (BD Biosciences; clone TRAP1), CD28-unlabeled (BD Biosciences; clone CD28.2), and CD49d-unlabeled (BD Biosciences; clone 9F10) for 18 hours, then stained with the following panel: CD3-APC-eFluor 780, CD4-BUV563, CD8-FITC, CD14-Alexa Fluor 700, CD19-Alexa Fluor 700, PDL1-PE, OX40-Pe-Cy7, CD137-BV650, CD69-BUV737, and PD1-BV785. CD4^+^ T-cell activation was determined by dual expression of OX40 and CD137.^36^ CD8^+^ T-cell activation was determined by dual expression of CD69 and CD137.^36,37^ For Intracellular Cytokine Staining (ICS), cells were cocultured with CD28-unlabeled (BD Biosciences; clone CD28.2), CD49d-unlabeled (BD Biosciences; clone 9F10), GolgiPlug (BD Biosciences), and GolgiStop (BD Biosciences) for 12 hours, then stained with the following panel: CD3-APC-eFluor 780, CD4-BUV563, CD8-V500, CD14-BV605, CD19-PerCP-Cy5.5, and Live/Dead-UV blue (Invitrogen). On completion, cells were fixed and permeabilized with Cytofix/Cytoperm (BD Biosciences) and intracellular stained with the following panel for 30 minutes: IL-17A-PE-eFluor 610, TNFα-PE-Cy7, IL-2-APC, and IFN-γ-Alexa Fluor 700. For AIM and ICS, gates were set by conducting negative and positive control tests as described previously (see Figure, Supplemental Digital Content 5, http://links.lww.com/QAI/C239, Figure, Supplemental Digital Content 6, http://links.lww.com/QAI/C240, Gating strategy).

### Statistics

Comparisons between groups (HIV + COVID vs COVID, hospitalized vs nonhospitalized) were tested for statistical significance using Mann–Whitney *U* tests. Comparisons based on hospitalization status were limited to the HIV + COVID group, which had a near equal representation of hospitalized and nonhospitalized cases. Linear regression modeling was used to determine whether significant relationships were explained by biases because of differences in age or hospitalization status. Participants were defined as having an AIM or ICS response if they met both of the following criteria: (1) The frequency of AIM/ICS-positive cells for the peptide stimulation was three-times higher than the frequency of AIM/ICS positive cells for the unstimulated control; (2) The number of AIM/ICS-positive cells for the peptide stimulation had a Fisher exact *P* value of < 0.0001 when compared with the unstimulated control; criteria adapted from flow cytometric optimization assays.^38^ For ICS, a participant was defined as a responder if they had a positive response for at least 1 cytokine. Comparisons with *P* values ≤ 0.05 were considered statistically significant. All statistical analysis was performed in R (v4.2.2 R Core Team 2022). All graphing was performed using GraphPad Prism version 9.3.1 (GraphPad Software).

## RESULTS

### Study Population Characteristics

Participants from both cohorts had similar sample collection time points, with the majority falling within 1-week postsymptom onset (HIV + COVID range 1–20 days; COVID range 0–16 days). Participants with chronic HIV infection (HIV + COVID) were younger than PWOH (*P* = 0.001). Most PWOH were hospitalized (n = 36, 88%), whereas 8 of 20 PWH were hospitalized (40%; *P* < 0.001) (Table 1). We controlled for cohort differences by performing generalized linear models comparing HIV + COVID and COVID groups while adjusting for age and hospitalization status (see Table, Supplemental Digital Content 7, http://links.lww.com/QAI/C241, Linear model). This analysis showed that the patterns of immune responses by HIV + COVID status did not seem to be driven by age or hospitalization status, indicated by the directionality of the beta-coefficients matching that of the unadjusted analyses. Observed differences between the HIV + COVID and COVID groups remained statistically significant after adjusting for age and hospitalization status unless otherwise noted.

### Monocyte and T-Cell Frequencies

**FIGURE 1.**
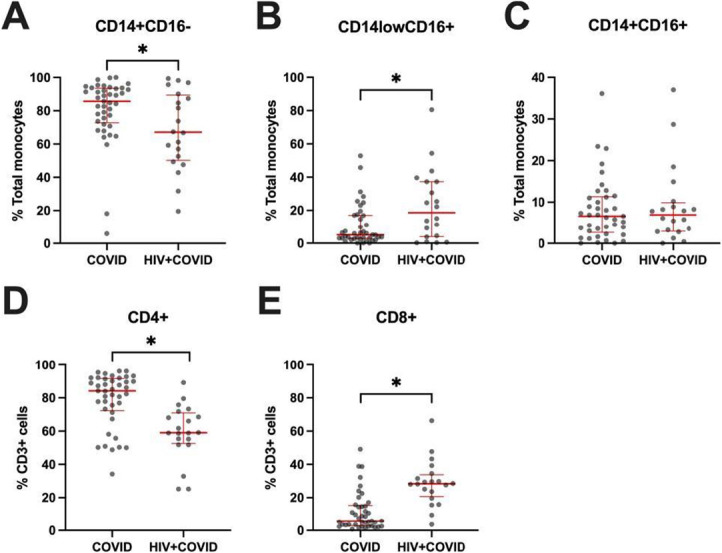
Monocyte and T-cell frequencies. Frequencies of A, classical, B, nonclassical, and C, intermediate monocytes. Frequencies of D, CD4^+^ and E, CD8^+^ T cells. COVID—PWOH experiencing acute COVID-19. HIV + COVID—HIV-positive participants experiencing acute COVID-19. Red bars denote median and interquartile range. *P* values determined by Mann–Whitney *U* test. *P ≤ 0.05.

We assessed immune cell population frequencies in participants with and without chronic HIV infection (Fig. 1). We first analyzed monocyte subsets because shifts in this population are associated with persistent inflammation in PWH and the hyperinflammatory state associated with severe acute COVID-19.^39–45^ PWH experiencing acute COVID-19 had lower frequencies of classical (CD14^+^) monocytes as compared to PWOH (*P* ≤ 0.05) (Fig. 1A). Relatedly, PWH experiencing acute COVID-19 had higher frequencies of nonclassical (CD16^+^) monocytes as compared to PWOH (*P* ≤ 0.05) (Fig. 1B). Frequencies of intermediate (CD14^+^CD16^+^) monocytes were similar between cohorts (Fig. 1C). We then analyzed T-cell frequencies, which are affected by both acute SARS-CoV-2 and chronic HIV infection.^7,46–59^ PWH had lower frequencies of CD4^+^ T cells and higher frequencies of CD8^+^ T cells compared with PWOH (both *P* ≤ 0.05) (Figs. 1D, E). We observed lower frequencies of CD38+HLA-DR+ CD4^+^ T cells in PWH compared with PWOH, although both groups had low frequencies of this immune cell subset, and no significant differences between groups were detected for other cell subsets (see Figure, Supplemental Digital Content 8, http://links.lww.com/QAI/C242, Immune cell populations).

### Activation and Exhaustion Markers

**FIGURE 2.**
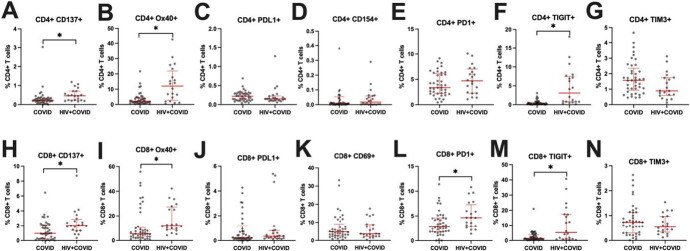
T-cell expression of activation and exhaustion markers. A–G, Frequencies of CD4^+^ T cells expressing activation markers CD137, OX40, PDL1, or CD154 or exhaustion markers PD1, TIGIT, or TIM3. H–N, Frequencies of CD8^+^ T cells expressing activation markers CD137, OX40, PDL1, or CD69 or exhaustion markers PD1, TIGIT, or TIM3. COVID—PWOH experiencing acute COVID-19. HIV + COVID—HIV-positive participants experiencing acute COVID-19. Red bars denote median and interquartile range. *P* values determined by Mann–Whitney *U* test. *P ≤ 0.05.

We next assessed expression of activation and exhaustion markers on CD4^+^ and CD8^+^ T cells to determine whether HIV infection altered their expression (Fig. 2). Compared with PWOH, PWH had increased expression of activation markers CD137 (Figs. 2A, H) and OX40 (Figs. 2B, I) and of exhaustion marker TIGIT (Figs. 2F, M) on CD4^+^ and CD8^+^ T cells (all *P* ≤ 0.05). PWH also had increased expression of exhaustion marker PD1 on CD8^+^ T cells (*P* ≤ 0.05) (Fig. 2L). No significant differences between the COVID and HIV + COVID cohorts were observed for other markers included in the panel.

### Immune Responses by Hospitalization Status

**FIGURE 3.**
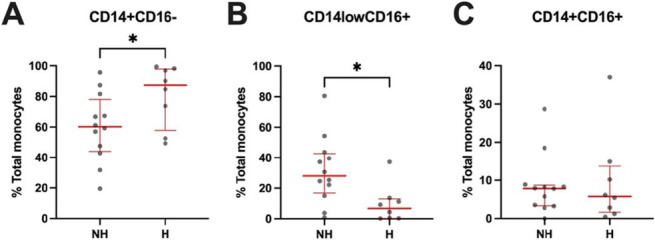
Monocyte frequencies from participants in the HIV + COVID cohort by hospitalization status. Frequencies of A, classical CD14^+^, B, nonclassical CD16^+^, and C, intermediate CD14^+^CD16^+^ monocytes. H, hospitalized; NH, nonhospitalized. Red bars denote median and interquartile range. *P* values determined by Mann–Whitney *U* test. *P ≤ 0.05.

**FIGURE 4.**
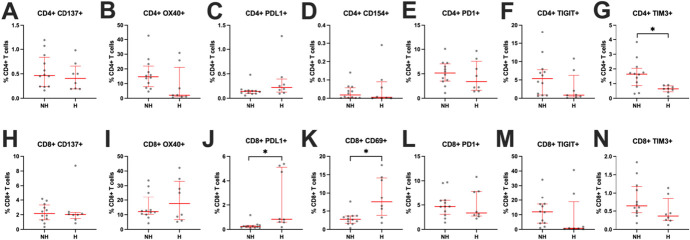
T-cell expression of activation and exhaustion markers from participants in the HIV + COVID cohort by hospitalization status. A–G, Frequencies of CD4^+^ T cells expressing activation markers CD137, OX40, PDL1, or CD154 or exhaustion markers PD1, TIGIT, or TIM3. H–N, Frequencies of CD8^+^ T cells expressing activation markers CD137, OX40, PDL1, or CD69 or exhaustion markers PD1, TIGIT, or TIM3. H, hospitalized; NH, nonhospitalized. Red bars denote median and interquartile range. *P* values determined by Mann–Whitney *U* test. *P ≤ 0.05.

We next assessed whether these immune findings were associated with clinical outcomes by stratifying our HIV + COVID group by hospitalization status. HIV-related characteristics were similar between hospitalized and nonhospitalized PWH (see Table, Supplemental Digital Content 1, http://links.lww.com/QAI/C235, HIV-related characteristics). All except 2 PWH—1 in each subgroup, both with viral loads < 500 copies/mL—had undetectable viral loads at the time of acute COVID-19. We first analyzed whether hospitalization was associated with changes in monocyte subset frequencies (Fig. 3). PWH who were hospitalized for acute COVID-19 had higher frequencies for classical monocytes (*P* ≤ 0.05) (Fig. 3A) and lower frequencies of nonclassical monocytes (*P* ≤ 0.05) (Fig. 3B). There were no differences in frequencies of intermediate monocytes between groups (Fig. 3C). We then analyzed whether hospitalization was associated with changes in expression of activation and exhaustion markers on CD4^+^ and CD8^+^ T cells (Fig. 4). PWH hospitalized for acute COVID-19 had decreased expression of TIM3 and trended toward decreased expression of OX40 on CD4^+^ T cells (*P* ≤ 0.05 and *P* = 0.057, respectively) (Figs. 4B, G). The hospitalized PWH group had increased expression of PDL1 and CD69 on CD8^+^ T cells (both *P* ≤ 0.05) (Figs. 4J, K). In contrast to these findings, previous studies in PWOH found heightened immune activation and exhaustion in those hospitalized for acute COVID-19.^6,26–34^ We also performed correlations between CD4% and our immune data to determine whether CD4% (13%–45%) influenced our findings and observed no significant relationships (see Table, Supplemental Digital Content 9, http://links.lww.com/QAI/C243, CD4% correlations), with the only exception a negative correlation with nonclassical monocytes (*P* = 0.030). There was no difference in CD4% by hospitalization status (nonhospitalized mean = 31, hospitalized mean = 33, *P* = 0.720).

### SARS-CoV-2–Specific Immune Responses

After characterizing the immune compartments during acute COVID-19 among our cohort, we analyzed SARS-CoV-2–specific immune responses through AIM and ICS assays (see Table, Supplemental Digital Content 10, http://links.lww.com/QAI/C244, Table, Supplemental Digital Content 11, http://links.lww.com/QAI/C245, Response frequencies). Previous research has demonstrated increased antigen-specific T-cell responses and decreased functionality in those with moderate-to-severe acute COVID-19 as early as 1-month postsymptom onset.^60,61^ When stimulated with either SARS-CoV-2 peptide pool, 2 (4.9%) and 4 (20%) of the COVID and HIV + COVID groups, respectively, had a CD4^+^ T-cell AIM response. There were no CD8^+^ T-cell AIM responses from either group. For ICS, 23 (56.1%) and 12 (60%) of the COVID and HIV + COVID groups, respectively, had a positive CD4^+^ T-cell response when stimulated with the SARS-CoV-2 S pool. Approximately 26 (63.4%) and 11 (55%) of the COVID and HIV + COVID groups, respectively, had a positive CD4^+^ T-cell response when stimulated with the SARS-CoV-2 M pool. There were few CD8^+^ T-cell ICS responses for either peptide pool among both cohorts. CD4^+^ T-cell ICS responses were primarily positive for TNF-α, with a few positive responses by intracellular IL-2 and IL-17A and no observed responses after IFN-γ. In summary, we detected limited SARS-CoV-2–specific responses in early infection with a functional bias toward TNF-α.

## DISCUSSION

As the COVID-19 pandemic continues to evolve, understanding factors that contribute to the development of severe disease is critical to improved clinical management and outcomes, particularly for at-risk populations such as PWH. Here, we evaluated participants within the first 21 days of symptom onset and confirmed SARS-CoV-2 infection, with or without chronic HIV. We sought to determine whether immune responses during acute SARS-CoV-2 infection differ in PWH as compared to PWOH. We also investigated how these shifts in immune response may relate to acute COVID-19 disease severity. To date, there are very limited data on early immunologic signatures in PWH.^25^ We hypothesized that PWH would have increased immune activation and exhaustion as compared to PWOH with acute COVID-19.

Looking first at cell population frequencies, we observed that PWH had lower frequencies of classical monocytes and higher frequencies of nonclassical monocytes during acute COVID-19. The cohort of PWH evaluated here was notable for consistent ART use and viral suppression. A higher prevalence of nonclassical and intermediate monocytes has been previously observed in chronic HIV infection and is associated with HIV disease progression.^45,62–64^ Thus, our finding of a negative correlation between CD4% and nonclassical monocyte frequency further corroborates that HIV infection may be driving the observed monocyte subset frequency changes when comparing PWH with PWOH.

When investigating T-cell populations, we observed PWH had increased expression of activation markers CD137 and OX40. Both activation markers have roles in enhancing T-cell survival, proliferation, differentiation, and effector functions. These markers also enhance T-cell function in latently HIV-infected T cells, thus contributing to persistent viral reservoirs.^34,65–71^ Our group and others have previously shown that PWOH with severe acute COVID-19 have increased expression of various activation markers on CD4^+^ and CD8^+^ T cells,^6,26–34^ indicating higher degrees of activation are associated with increased disease severity. We also observed PWH had increased expression of exhaustion markers PD1 and TIGIT on both CD4^+^ and CD8^+^ T cells as compared to PWOH. Both PD1 and TIGIT are exhaustion markers associated with immune dysfunction and have previously been reported in HIV disease progression.^32,72–79^ Our laboratory previously observed PD1 expression to be increased on CD4^+^ and CD8^+^ T cells in patients hospitalized from acute COVID-19 and found TIGIT expression to be increased on CD4^+^ and CD8^+^ T cells during acute COVID-19, regardless of disease severity.^34^ Only 2 previous studies have evaluated PD1 expression in PWH with acute COVID-19 and found its expression to be increased compared with PWOH.^23,25^ However, the expression of PD1 was only studied while coexpressed with either HLA-DR^23^ or TIM3^25^ in these studies. Notably, the expression of T-cell activation and exhaustion markers CD137, OX40, and TIGIT have not been previously reported in PWH with acute COVID-19. The broad immune activation and exhaustion observed in the HIV + COVID cohort compared with the older PWOH with more severe disease suggests that chronic HIV infection substantially augments the immune response in PWH, despite effective viral suppression by ART.^23,25,72–74^

We hypothesized that immune dysregulation would be further amplified in severe presentations of acute COVID-19 in PWH. PWH who were hospitalized had higher frequencies of classical monocytes and lower frequencies of nonclassical monocytes, which aligns with previous reports in PWOH.^41,43,44^ During severe acute COVID-19, excess inflammation contributes to lung damage and increased oxygenation requirements. This hyperinflammatory state is partially driven by classical monocytes, which contribute to the production of proinflammatory cytokines such as IL-1β, IL-6, and TNF-α.^51,52,58^ Our finding of increased classical monocytes in hospitalized PWH suggests that, similar to PWOH, classical monocytes contribute to more severe presentations of acute COVID-19 in PWH. The role of nonclassical monocytes is less clear and depends on an individual's baseline immunological profile. During severe acute COVID-19, nonclassical monocytes are decreased during severe acute COVID-19, which could be due to migration of this subset to inflamed tissues such as the lung.^80,81^ By contrast, chronic HIV infection is associated with immune dysfunction, including expansion of nonclassical monocytes and a shift in this subset toward a more proinflammatory phenotype.^45,62–64^ Further research is required to fully understand the role of monocytes in PWH infected with acute COVID-19, as well as how different monocyte subsets interact and affect other innate and adaptive immune cells.

We also observed decreased expression of TIM3 on CD4^+^ T cells and increased expression of activation markers PDL1 and CD69 on CD8^+^ T cells. Similar to PD1 and TIGIT, TIM3 is an exhaustion marker known to be elevated in chronic viral infections such as HIV and correlates with HIV disease progression.^76–78,82^ Our group has previously shown increased expression of TIM3 on T cells from PWOH hospitalized for acute COVID-19.^34^ To the best of our knowledge, there has only been 1 previous report exploring TIM3 expression in PWH hospitalized for COVID-19, which demonstrated elevations in exhausted T cells coexpressing TIM3 and PD1 in PWH compared with PWOH hospitalized acute COVID-19.^25^ In contrast to our observations between hospitalized and nonhospitalized PWH, previous reports in PWOH show increased expression of various T-cell activation and exhaustion markers in those hospitalized for acute COVID-19 compared with nonhospitalized individuals.^6,26–34^ This suggests that there may be other underlying factors driving COVID-19 disease severity in PWH such as innate immune responses, pre-existing cross-reactive humoral immune responses, and metabolic derangements.

As part of our analysis, we performed SARS-CoV-2–specific assays to determine whether there was a difference in antigen-specific responses based on HIV status. To reduce confounding variables, we limited our study to unvaccinated individuals with no documented history of SARS-CoV-2 infection. We noted only a limited number of antigen-specific responses overall. Development of CD4^+^ and CD8^+^ T-cell responses to infections is often detected as early as one week after infection but usually peak at around 2 weeks after infection.^83–86^ Most participants within our study had samples collected within the first week of symptom onset, which likely explains the low frequency of antigen-specific responses. Although most participants did not have measurable ICS responses for IL-2 and IFN-γ, more than half of participants from both the COVID and HIV + COVID cohorts had responses for TNF-α. Others have reported similar findings of an impaired antiviral response during acute COVID-19.^87,88^ Galani et al^89^ suggested that this impairment could be due to timing of sample collection and determined that, unlike other viral infections, SARS-CoV-2 infection leads to a proinflammatory response before interferon-mediated antiviral responses.

We crafted our cohort based on sex and race, which led to differences in age and acute disease severity between our groups. Because of the small sample size of hospitalized PWH and nonhospitalized PWOH, and significant differences in age between our groups, we used general linear modeling to adjust for these potential confounders. We found that all significant relationships with immune responses as determined by Mann–Whitney *U* tests had the same associations with HIV + COVID status after adjusting for age and hospitalization, indicating that these characteristics were not driving the observed differences between the HIV + COVID and COVID groups. Despite our PWOH group exhibiting more severe disease and older age, the HIV + COVID cohort exhibited increased levels of immune activation and exhaustion, emphasizing the impact that HIV infection has on T-cell immune responses. Furthermore, our study only included samples from participants who were likely infected with the Alpha SARS-CoV-2 variant based on timing of infection. Future studies could benefit from analyzing patients with more recent variants such as Omicron to assess how different variants and baseline immunity may affect immune responses. There was only 1 sample collection timepoint for all participants, with most timepoints falling within 1 week of symptom onset, thereby limiting our temporal assessment of the immune response during acute COVID-19.

In summary, our findings demonstrate acute SARS-CoV-2 infection in PWH leads to heightened immune activation as compared to PWOH. Notably, these observations were apparent despite older age and increased COVID-19 severity in the PWOH, highlighting the drastic impact chronic HIV infection has on the acute immune response to SARS-CoV-2. We observed lower frequencies of nonclassical monocytes in hospitalized PWH and few differences in T-cell activation and exhaustion marker expression based on hospitalization status in PWH. Assessing how other viral pathogens may differentially affect at-risk populations, particularly in PWH where a baseline increase in inflammation persists, warrants additional study. Here, our findings provide rationale for future large-scale studies of SARS-CoV-2 in PWH and other conditions resulting in chronic immune dysregulation and may lead to the identification of potential immunotherapy targets to alleviate symptomatic burden.

## Supplementary Material
