# Supplementary material for: Impact of Chronic HIV Infection on Acute Immune Responses to SARS-CoV-2
Source: J Acquir Immune Defic Syndr. 2024 Feb 26;96(1):92–100. doi: 10.1097/QAI.0000000000003399 (PMC11009054; doi:10.1097/QAI.0000000000003399)
Supplement: Supplementary file 1 [file qai-96-92-s001.docx]

**Supplemental Digital Content 1. HIV-related characteristics by COVID-19 hospitalization status for people with HIV (n=20)**

|  | **Non-hospitalized** | **Hospitalized** | **p value** |
| --- | --- | --- | --- |
| **Sample size** | 12 | 8 |  |
| **Years since HIV diagnosis ^A^** | 18 (4-40) | 17 (8-28) | 0.894 |
| **Prior AIDS ^B^** | 2 (17%) | 3 (38%) | 0.344 |
| **Viral load < 200 copies/mL ^B^** | 11 (92%) | 7 (88%) | > 0.999 |
| **CD4 - current ^A^** |  |  |  |
| Count | 750 (266-1619) | 698 (181-1312) | 0.792 |
| Percent | 31 (13-45) | 33 (16-45) | 0.720 |
| **CD4 - nadir ^A^** |  |  |  |
| Count | 19 (4-37) | 16 (4-35) | 0.585 |
| Percent | 330 (39-958) | 233 (16-424) | 0.664 |
| **Comorbidities ^B^** |  |  |  |
| Hypertension | 9 (75%) | 7 (88%) | 0.619 |
| Obesity | 5 (48%) | 4 (50%) | > 0.999 |
| Diabetes | 2 (17%) | 5 (63%) | 0.062 |
| COPD | 2 (17%) | 4 (50%) | 0.161 |
| Cancer | 2 (17%) | 2 (25%) | > 0.999 |
| **ART Regimen ^B^** |  |  |  |
| NRTI | 11 (92%) | 8 (100%) | > 0.999 |
| NNRTI | 0 (0%) | 0 (0%) | > 0.999 |
| PI | 3 (25%) | 1 (13%) | 0.619 |
| INSTI | 10 (83%) | 8 (100%) | 0.495 |

Letters denote which statistical analysis was performed for each category: A – Mann Whitney U test, B – Fisher’s exact test. NRTI – nucleoside reverse transcriptase inhibitor, NNRTI – non-nucleoside reverse transcriptase inhibitor, PI – protease inhibitor, INSTI – integrase strand transfer inhibitor. Percentages were rounded to the nearest whole number.
