# Supplementary material for: Impact of Chronic HIV Infection on Acute Immune Responses to SARS-CoV-2
Source: J Acquir Immune Defic Syndr. 2024 Feb 26;96(1):92–100. doi: 10.1097/QAI.0000000000003399 (PMC11009054; doi:10.1097/QAI.0000000000003399)
Supplement: Supplementary file 2 [file qai-96-92-s002.docx]

**Supplemental Digital Content 2. Flow cytometry panels**

| **Panel** | **Fluorophore** | **Conjugate** | **Clone** | **Manufacturer** |
| --- | --- | --- | --- | --- |
| Immune cell populations | APC-eFluor 780 | CD3 | UCHT1 | eBioscience |
|  | BV711 | CD4 | SK3 | BD Biosciences |
|  | V500 | CD8 | RPA-T8 | BD Biosciences |
|  | Alexa Fluor 700 | CD14 | M5E2 | BD Biosciences |
|  | FITC | CD16 | eBioCB16 | eBioscience |
|  | BUV563 | CD19 | SJ25C1 | BD Biosciences |
|  | PE/Dazzle 594 | CD27 | O323 | BioLegend |
|  | APC | CD28 | CD28.2 | BD Biosciences |
|  | BUV737 | CD38 | HB7 | BD Biosciences |
|  | Pe-Cy7 | CD45 | HI30 | BD Biosciences |
|  | BV421 | CD56 | NCAM16.2 | BD Biosciences |
|  | PerCp-Cy5.5 | CD57 | HNK-1 | BioLegend |
|  | PE | HLA-DR | L243 or G46-6 | BD Biosciences |
| Activation and Exhaustion | APC-eFluor 780 | CD3 | UCHT1 | eBioscience |
|  | PE-eFluor 610 | CD4 | RPA-T4 | eBioscience |
|  | FITC | CD8 | SK1 | BD Biosciences |
|  | BUV563 | CD14 | MφP9 | BD Biosciences |
|  | BUV563 | CD19 | SJ25C1 | BD Biosciences |
|  | PeCy7 | OX40 | Ber-ACT35 | BioLegend |
|  | BUV737 | CD69 | FN50 | BD Biosciences |
|  | BV650 | CD137 | 4B4-1 | BD Biosciences |
|  | APC | CD154 | TRAP1 | BD Biosciences |
|  | BV785 | PD1 | EH12.2H7 | BioLegend |
|  | Alexa Fluor 700 | TIGIT | 741182 | R&D Systems |
|  | PE | PDL1 | MIH1 | BD Biosciences |
|  | BV421 | TIM3 | F38-2E2 | BioLegend |
| AIM | APC-eFluor 780 | CD3 | UCHT1 | eBioscience |
|  | BUV563 | CD4 | SK3 | BD Biosciences |
|  | FITC | CD8 | SK1 | BD Biosciences |
|  | Alexa Fluor 700 | CD14 | M5E2 | BD Biosciences |
|  | Alexa Fluor 700 | CD19 | HIB19 | BD Biosciences |
|  | PE | PDL1 | MIH1 | BD Biosciences |
|  | Pe-Cy7 | OX40 | Ber-ACT35 | BioLegend |
|  | BV650 | CD137 | 4B4-1 | BD Biosciences |
|  | BUV737 | CD69 | FN50 | BD Biosciences |
|  | APC | CD154 | TRAP1 | BD Biosciences |
|  | BV785 | PD1 | EH12.2H7 | BioLegend |
| ICS | APC-eFluor 780 | CD3 | UCHT1 | eBioscience |
|  | BUV563 | CD4 | SK3 | BD Biosciences |
|  | V500 | CD8 | RPA-T8 | BD Biosciences |
|  | BV605 | CD14 | M5E2 | BD Biosciences |
|  | PerCP-Cy5.5 | CD19 | SJ25C1 | BD Biosciences |
|  | PE-eFluor 610 | IL-17A | eBio64DEC17 | eBioscience |
|  | Pe-Cy7 | TNFα | MAB11 | BD Biosciences |
|  | APC | IL2 | 5344.111 | BD Biosciences |
|  | Alexa Fluor 700 | IFN-γ | B27 | BD Biosciences |
