# Supplementary material for: Impact of Chronic HIV Infection on Acute Immune Responses to SARS-CoV-2
Source: J Acquir Immune Defic Syndr. 2024 Feb 26;96(1):92–100. doi: 10.1097/QAI.0000000000003399 (PMC11009054; doi:10.1097/QAI.0000000000003399)
Supplement: Supplementary file 3 [file qai-96-92-s003.docx]

**Supplemental Digital Content 7. Linear regression model results for monocyte subsets and T cell activation and exhaustion markers in HIV+COVID and COVID participants**

|  |  | HIV+COVID status | | Increasing Age | | Hospitalization | |
| --- | --- | --- | --- | --- | --- | --- | --- |
| Subset | Marker | β | P value | β | P value | β | P value |
| Monocyte | CD14+CD16- | -11.57 | 0.08 | -0.35 | 0.06 | 10.37 | 0.13 |
|  | CD14lowCD16+ | 9.67 | **0.05*** | 0.28 | **0.05*** | -11.97 | **0.02*** |
| CD4 | -- | -14.38 | **0.01*** | 0.06 | 0.70 | 9.43 | 0.09 |
|  | CD137 | 0.15 | 0.29 | 0.00 | 0.47 | -0.02 | 0.91 |
|  | OX40 | 6.69 | **0.01*** | 0.02 | 0.75 | -6.85 | **0.01*** |
|  | TIGIT | 3.69 | **<0.01*** | 0.03 | 0.27 | -1.74 | 0.07 |
| CD8 | -- | 12.63 | **<0.01*** | -0.08 | 0.44 | -7.94 | **0.05*** |
|  | CD137 | 0.95 | 0.06 | -0.01 | 0.49 | -0.12 | 0.81 |
|  | OX40 | 1.78 | 0.69 | -0.06 | 0.61 | -6.18 | 0.17 |
|  | PD1 | 1.39 | 0.10 | 0.03 | 0.26 | -1.03 | 0.23 |
|  | TIGIT | 8.14 | **<0.01*** | 0.05 | 0.49 | -1.75 | 0.48 |

Beta coefficients (β) and p values are shown for each covariate included in the linear regression models. The beta coefficients for HIV+COVID status are interpretable as the difference in the cell subset frequency between participants with and without HIV infection or hospitalization, adjusting for age and hospitalization. Similarly, the beta coefficients for age are interpretable as the differences in cell subset frequency for a 1-year greater age, adjusted for HIV and hospitalization, and the beta coefficients for hospitalization are interpretable as the differences in cell subset frequency between people who were and were not hospitalized, adjusted for HIV and age. P values ≤ 0.05 were considered to be statistically significant and are indicated in bold.
