# Supplementary material for: Impact of Chronic HIV Infection on Acute Immune Responses to SARS-CoV-2
Source: J Acquir Immune Defic Syndr. 2024 Feb 26;96(1):92–100. doi: 10.1097/QAI.0000000000003399 (PMC11009054; doi:10.1097/QAI.0000000000003399)
Supplement: Supplementary file 4 [file qai-96-92-s004.docx]

**Supplemental Digital Content 9. Correlations between CD4% and immune cell subsets**

| Subset | Marker | Coefficient | P value |
| --- | --- | --- | --- |
| Monocyte | CD14+CD16- | 0.265 | 0.259 |
|  | CD14-CD16+ | -0.484 | **0.030*** |
|  | CD14+CD16+ | 0.302 | 0.196 |
| CD4 | CD137 | -0.363 | 0.115 |
|  | OX40 | 0.109 | 0.647 |
|  | PDL1 | -0.145 | 0.542 |
|  | CD154 | -0.348 | 0.132 |
|  | PD1 | -0.242 | 0.305 |
|  | TIGIT | -0.145 | 0.543 |
|  | TIM3 | 0.148 | 0.533 |
|  | CD137 | -0.036 | 0.880 |
|  | OX40 | 0.084 | 0.724 |
|  | PDL1 | -0.112 | 0.637 |
| CD8 | CD69 | 0.130 | 0.586 |
|  | PD1 | -0.091 | 0.702 |
|  | TIGIT | 0.132 | 0.580 |
|  | TIM3 | 0.002 | 0.992 |

Coefficients and p values are shown for each correlation performed. P values ≤ 0.05 were determined to be statistically significant.
