# Supplementary material for: Impact of Chronic HIV Infection on Acute Immune Responses to SARS-CoV-2
Source: J Acquir Immune Defic Syndr. 2024 Feb 26;96(1):92–100. doi: 10.1097/QAI.0000000000003399 (PMC11009054; doi:10.1097/QAI.0000000000003399)
Supplement: Supplementary file 5 [file qai-96-92-s005.docx]

**Supplemental Digital Content 10. AIM response frequencies**

|  | **HIV+COVID** | **COVID** | **p value** |
| --- | --- | --- | --- |
| **S pool** |  |  |  |
| CD4+OX40+CD137 | 4 (20%) | 2 (4.9%) | 0.084 |
| CD8+CD69+CD137 | 0 (0%) | 0 (0%) | 1 |
| **M pool** |  |  |  |
| CD4+OX40+CD137 | 4 (20%) | 2 (4.9%) | 0.084 |
| CD8+CD69+CD137 | 0 (0%) | 0 (0%) | 1 |

P values were calculated using Fisher’s exact test.
