# Supplementary material for: Impact of Chronic HIV Infection on Acute Immune Responses to SARS-CoV-2
Source: J Acquir Immune Defic Syndr. 2024 Feb 26;96(1):92–100. doi: 10.1097/QAI.0000000000003399 (PMC11009054; doi:10.1097/QAI.0000000000003399)
Supplement: Supplementary file 6 [file qai-96-92-s006.docx]

**Supplemental Digital Content 11. ICS response frequencies**

|  | **HIV+COVID** | **COVID** | **p value** |
| --- | --- | --- | --- |
| **S pool** |  |  |  |
| CD4+ IFN-g | 0 (0%) | 0 (0%) | 1 |
| CD4+ IL-2 | 5 (25%) | 2 (4.9%) | **0.033*** |
| CD4+ IL-17A | 0 (0%) | 1 (2.4%) | 1 |
| CD4+ TNF-a | 9 (45%) | 23 (56.1%) | 0.586 |
| CD4+ ICS total | 12 (60%) | 23 (56.1%) | 1 |
| CD8+ IFN- g | 1 (5%) | 1 (2.4%) | 1 |
| CD8+ IL-2 | 2 (10%) | 0 (0%) | 0.104 |
| CD8+ TNF-a | 3 (15%) | 0 (0%) | **0.032*** |
| CD8+ ICS total | 4 (20%) | 1 (2.4%) | **0.036*** |
| **M pool** |  |  |  |
| CD4+ IFN- g | 0 (0%) | 0 (0%) | 1 |
| CD4+ IL-2 | 4 (20%) | 4 (9.8%) | 0.420 |
| CD4+ IL-17A | 2 (10%) | 4 (9.8%) | 1 |
| CD4+ TNF-a | 9 (45%) | 24 (58.5%) | 0.414 |
| CD4+ ICS total | 11 (55%) | 26 (63.4%) | 0.584 |
| CD8+ IFN- g | 0 (0%) | 0 (0%) | 1 |
| CD8+ IL-2 | 0 (0%) | 0 (0%) | 1 |
| CD8+ TNF-a | 1 (5%) | 0 (0%) | 0.328 |
| CD8+ ICS total | 1 (5%) | 0 (0%) | 0.328 |

P values were calculated using Fisher’s exact test.
