# Supplementary material for: Impact of Chronic HIV Infection on Acute Immune Responses to SARS-CoV-2
Source: J Acquir Immune Defic Syndr. 2024 Feb 26;96(1):92–100. doi: 10.1097/QAI.0000000000003399 (PMC11009054; doi:10.1097/QAI.0000000000003399)
Supplement: Supplementary file 9 [file qai-96-92-s009.pdf]

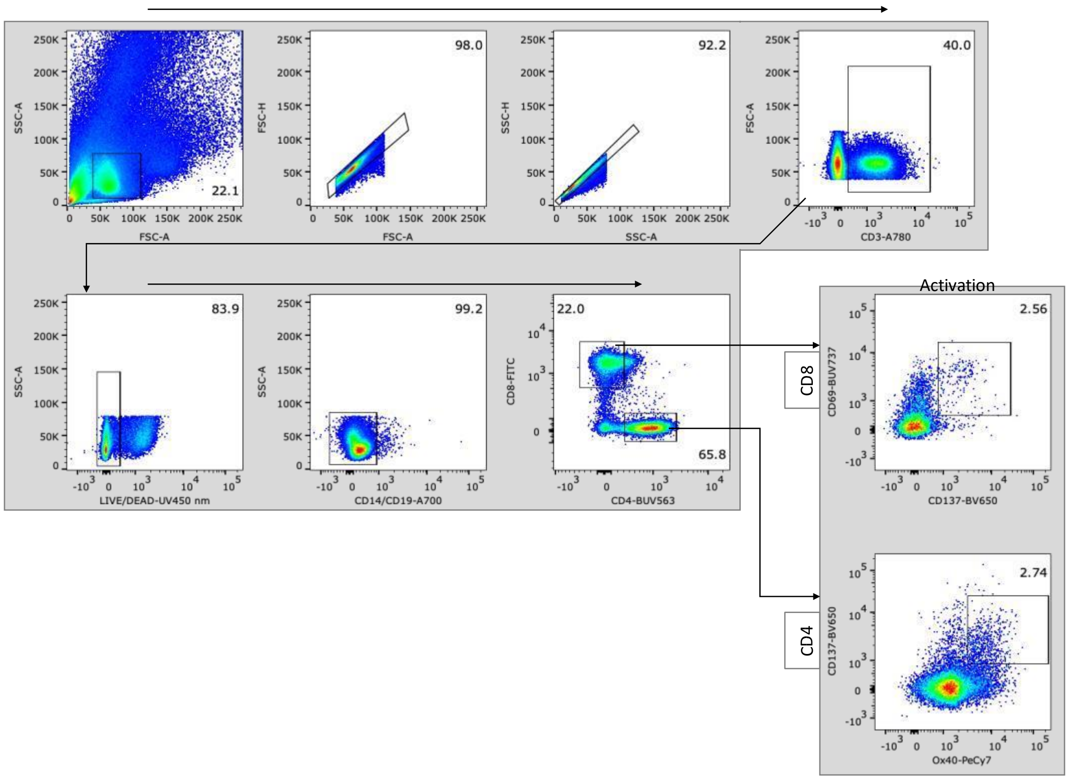

Supplemental Digital Content 5. Gating strategy for AIM panel. Approach for identifying SARS-CoV-2-specific T cells.
