# Supplementary material for: Impact of Chronic HIV Infection on Acute Immune Responses to SARS-CoV-2
Source: J Acquir Immune Defic Syndr. 2024 Feb 26;96(1):92–100. doi: 10.1097/QAI.0000000000003399 (PMC11009054; doi:10.1097/QAI.0000000000003399)
Supplement: Supplementary file 11 [file qai-96-92-s011.pdf]

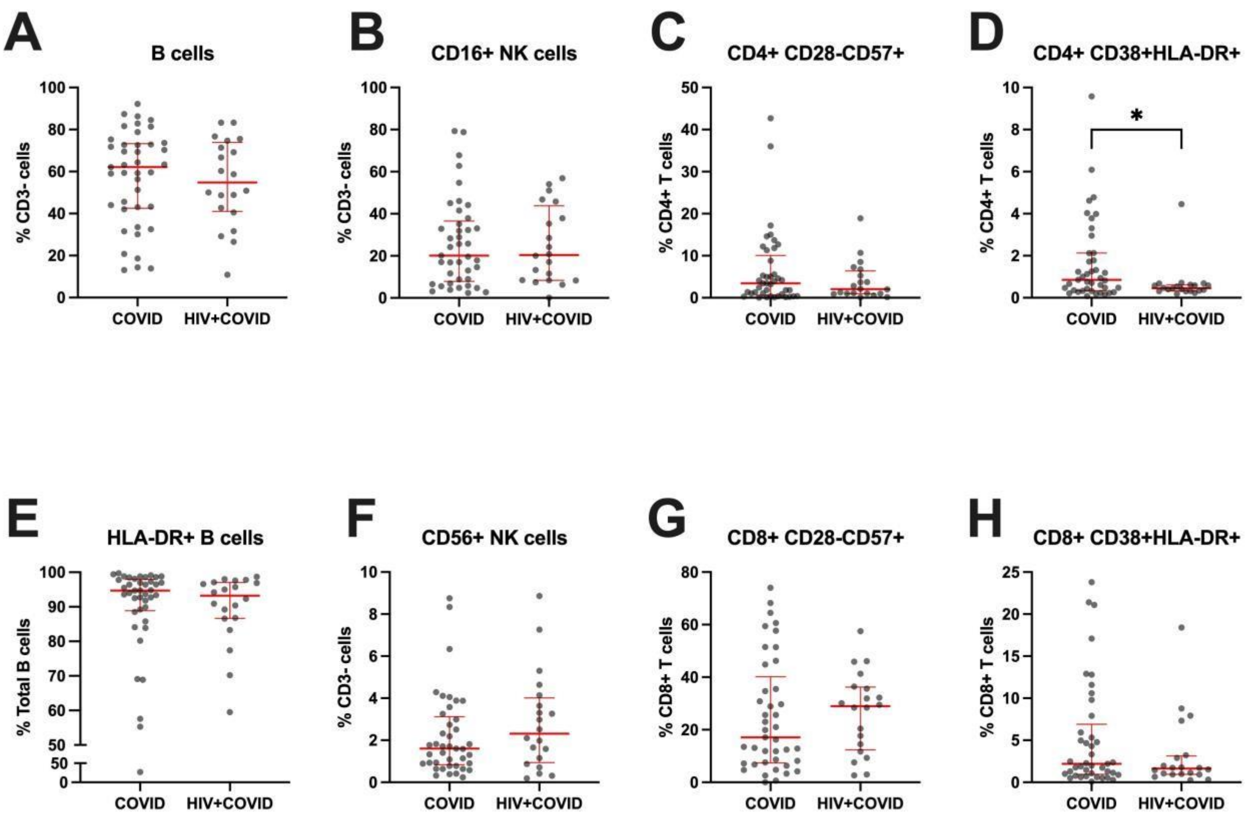

Supplemental Digital Content 8. Immune cell populations. Frequencies of B cells, NK cells, and CD4+ and CD8+ T cells. COVID – HIV-uninfected participants experiencing acute COVID-19. HIV+COVID – HIV-positive participants experiencing acute

COVID-19. Red bars denote median and interquartile range. P values determined by Mann Whitney U test.* p 0.05
