# Supplementary material for: Impact of Chronic HIV Infection on Acute Immune Responses to SARS-CoV-2
Source: J Acquir Immune Defic Syndr. 2024 Feb 26;96(1):92–100. doi: 10.1097/QAI.0000000000003399 (PMC11009054; doi:10.1097/QAI.0000000000003399)
Supplement: Supplementary file 7 [file qai-96-92-s007.pdf]

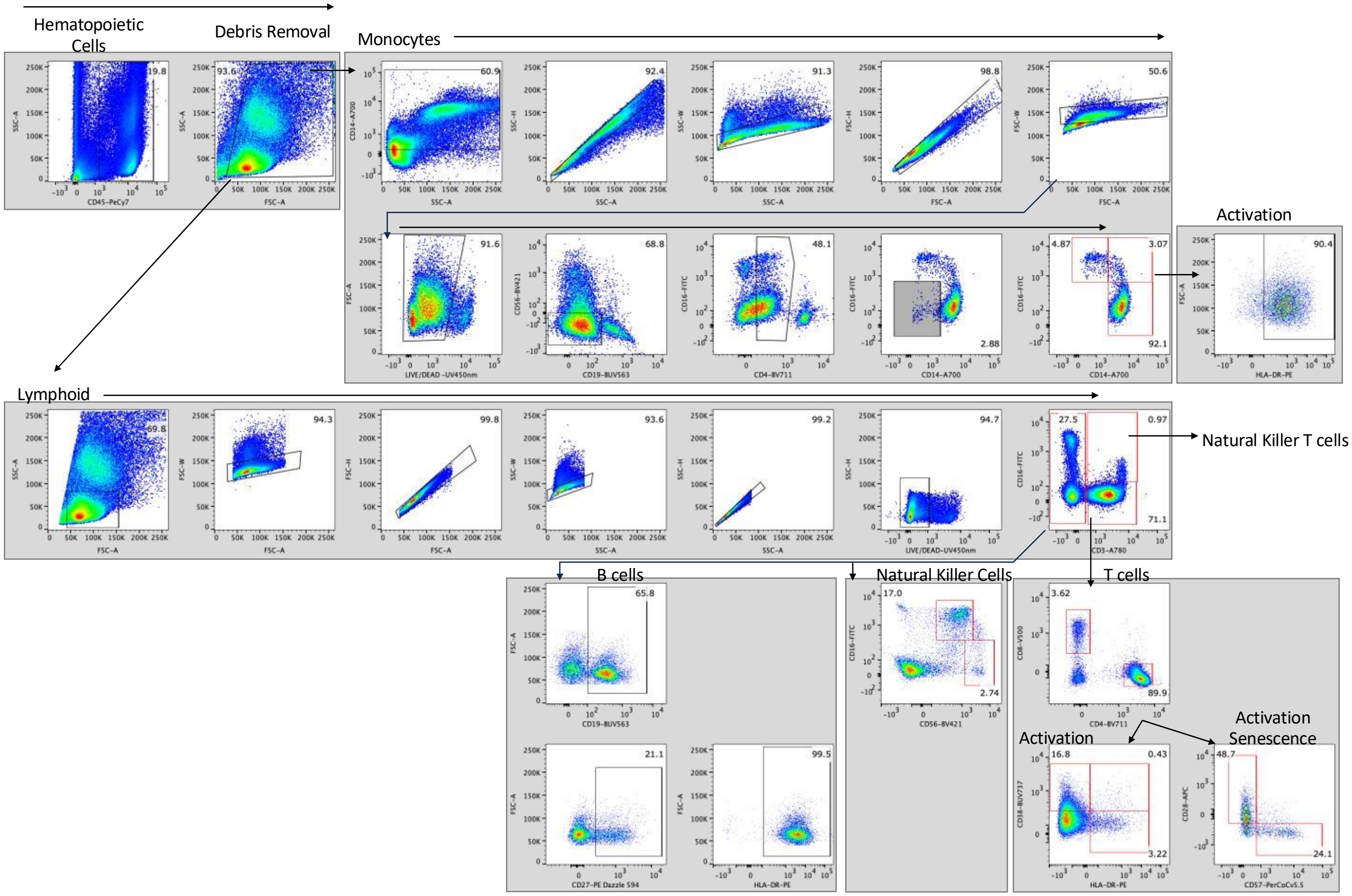

Supplemental Digital Content 3. Gating strategy for Immune cell population panel. Approach for identifying monocytes, NK cells, B cells, and T cells.
